# Impacts of urban green infrastructure on attentional functioning: insights from an fMRI study

**DOI:** 10.3389/fpsyg.2023.1047993

**Published:** 2023-05-23

**Authors:** Xiangrong Jiang, Yifan Hu, Linda Larsen, Chun-Yen Chang, William C. Sullivan

**Affiliations:** ^1^School of Architecture and Civil Engineering, Xihua University, Chengdu, China; ^2^Department of Psychology, University of Illinois at Urbana-Champaign, Champaign, IL, United States; ^3^Department of Landscape Architecture, University of Illinois at Urbana-Champaign, Champaign, IL, United States; ^4^Department of Horticulture and Landscape Architecture, National Taiwan University, Taipei, Taiwan

**Keywords:** green infrastructure, trees, bioswales, attention restoration, fMRI, functional connectivity

## Abstract

Multiple studies using various measures, technologies, and participant groups have found that exposure to urban green infrastructure can help alleviate the daily attentional fatigue that human experience. Although we have made significant progress in understanding the effects of exposure to urban green infrastructure on attention restoration, two important gaps in our knowledge remain. First, we do not fully understand the neural processes underlying attention restoration that exposure to urban green infrastructure elicits. Second, we are largely unaware of how typical patterns of urban green infrastructure, such as combinations of trees and bioswales, affect recovery from attentional fatigue. This knowledge is crucial to guide the design and management of urban landscapes that effectively facilitate attention restoration. To address these gaps in our knowledge, we conducted a controlled experiment in which 43 participants were randomly assigned to one of three video treatment categories: no green infrastructure (No GI), trees, or trees and bioswales. We assessed attentional functioning using functional Magnetic Resonance Imaging (fMRI) and the Sustained Attention Response Task (SART). Participants exposed to urban settings with trees exhibited improved top-down attentional functioning, as evidenced by both fMRI and SART results. Those exposed to urban settings with trees and bioswales demonstrated some attention-restorative neural activity, but without significant improvements in SART performance. Conversely, participants exposed to videos of urban environments without green infrastructure displayed increased neural vigilance, suggesting a lack of attention restoration, accompanied by reduced SART performance. These consistent findings offer empirical support for the Attention Restoration Theory, highlighting the effectiveness of tree exposure in enhancing attentional functioning. Future research should investigate the potential impact of bioswales on attention restoration.

## 1. Introduction

Effective functioning requires the ability to focus one’s attention. Top-down attention, which enables organized and purposeful thinking, is essential for success in life (Sullivan and Li, 2021; Jiang et al., 2022). This kind of attention allows people to focus on current tasks and resist external distractions (Kaplan and Berman, 2010). Top-down attention enables planning, organization, and completion of tasks that involve conflicts (Fan et al., 2002), the achievement of high-level goals (Kuo, 2001; Asplund et al., 2010), and the detection of targets (Dichter et al., 2009). Top-down attention, however, easily becomes fatigued and requires regular rest to be restored.

Previous research has demonstrated that top-down attention can be restored through exposure to nature (Berto, 2005; Berman et al., 2008; Jiang et al., 2019). These findings align with Attention Restoration Theory (Kaplan, 1995), which posits that exposure to nature restores top-down attention by utilizing bottom-up attention, or automatic attention triggered by external stimuli, thus allowing top-down attention a chance to rest and recover.

Urban residents, particularly those living in high-density areas, often have limited access to nature (Burgess, 2000; Ahern, 2007; United Nations Centre for Human Settlements, 2012). Urban green infrastructure (UGI), including urban parks, pocket parks, trees, bioswales, and roof gardens, serve as the primary source of nature for city dwellers. Although research acknowledges the potential of green infrastructure (GI) to restore attention (Tzoulas et al., 2007), most studies have focused on the impact of trees, parks, green spaces, and urban forests on top-down attention (Taylor et al., 2002; Nordh et al., 2011; Shin et al., 2011; Yin et al., 2020). The effects of other types of GI, such as bioswales, on attentional fatigue recovery remain unexamined. While previous research has demonstrated the beneficial relationship between exposure to certain types of GI and attentional functioning, much of this research has relied on attentional tests or self-reported questionnaires. Some studies have also utilized brain imaging techniques to observe the effects of nature and built environment images on brain activity (Kim G.-W. et al., 2010; Kuhn et al., 2021), but these studies do not specifically examine the effects of urban GI.

This gap in knowledge limits our understanding of the neural mechanisms underlying attention restoration from exposure to various forms of green infrastructure and the landscape characteristics that promote attention restoration. The study presented here aims to address these gaps in knowledge. We begin by reviewing the theory and evidence concerning the impact of green infrastructure on attention restoration, followed by an examination of the literature on neural pathways involved in attentional functioning. Next, we present the results of a randomized controlled experiment on the effects of urban green infrastructure on attentional functioning, including novel findings on the extent of neural connectivity related to attention fostered by exposure to green infrastructure. Finally, we will discuss the contributions of this work, the implications of the findings, and directions for future research.

## 2. Attention restoration theory and empirical evidence

Attention Restoration Theory (ART) posits that exposure to natural environments can restore an individual’s top-down attention, or the capacity to focus on a single task while ignoring distractions (Kaplan, 1995). According to ART, a restorative environment should allow an individual to feel like they are away from their daily routine, provide “soft fascination” that attracts attention effortlessly, offer a sense of extent that is rich and coherent enough to engage the mind, and be compatible with the individual’s goals (Sullivan and Li, 2021). Natural environments with these four characteristics are unlikely to engage top-down attention. That is, natural features hold people’s attention through a bottom-up attentional process that allows top-down attention to rest and restore (Berman et al., 2008). Bottom-up attention, which is effortless, acts like a circuit breaker to interrupt top-down attentional functioning (Corbetta and Shulman, 2002).

Aside from natural stimuli, various other stimuli can interrupt top-down attention, think of, for instance a fire alarm in a library or social interactions. Although these stimuli trigger bottom-up attention, − the mental response to the alarm is automatic – they do not offer restorative benefits because they require top-down attention to determine a course of action, evoking hard rather than soft fascination which reengages top-down attention (Sullivan and Li, 2021).

Numerous studies have documented the attentional functioning benefits that result from exposure to nature (Norwood et al., 2019; Jiang et al., 2020; Buttazzoni et al., 2021). University students who, for example, live in dorms with natural views scored better on tests of attention than did those without green views (Tennessen and Cimprich, 1995). An experimental study demonstrated that walking for 20 min in a park boosted children’s attention performance (Taylor and Kuo, 2009). Similarly, women who were diagnosed with breast cancer achieved better attention restoration when they were exposed to nature for only 120 min per week (Cimprich and Ronis, 2003). Such results, which involve people of different ages and in various degrees of health, provide solid external validity to ART.

Previous studies have measured attentional functioning through questionnaires or behavior tests and have established a positive relationship between exposure to nature and attentional functioning – generally, greater exposure leads to better attentional functioning (Sullivan and Li, 2021). These studies have focused primarily on urban tree canopy and urban parks. Little is known about the impact of other forms of green infrastructure, such as bioswales, on attention. The impact of other green infrastructure types, such as bioswales, on attention remains underexplored. Bioswales, a typical form of UGI, address urban stormwater challenges by capturing and filtering runoff before it enters local water supplies or sewer systems. Many cities are increasingly adopting ecological grass swales to combat urban flooding (Qin, 2020). However, little is known about bioswales’ effects on attentional function. Moreover, researchers have only just begun to observe the neural activities stimulated by exposure to natural and built environments. To our knowledge, no studies have examined the impact of the newer types of green infrastructure on neural activities associated with attention in urban contexts.

## 3. Brain activities of attentional functioning and restoration

As the scope and complexity of attentional functioning research grows, so does the emergence of new domains with more diverse, frequently transdisciplinary research approaches. Neurourbanism, an interdisciplinary approach that connects neuroscience, public health, epidemiology, landscape architecture, urban planning and other relevant subjects with the objective of constructing more healthy places, is one such progression (Adli et al., 2017). Researchers have documented the relationship between urban environments and brain activities (Buttazzoni et al., 2021). Two studies, for instance, have explored brain activation patterns while participants were viewing natural and urban scenery (Kim G.-W. et al., 2010; Kim T.-H. et al., 2010; Norwood et al., 2019).

Functional Magnetic Resonance Imaging (fMRI) technology has also been used to explored the neural activities that grow from exposure to various landscape environments (Tang et al., 2017). One study using fMRI technology, reported that when participants viewed images of nature, their neural activity was associated with bottom-up attention, but when they viewed images of buildings that did not contain any vegetation, their neural activity was associated with top-down attention (Martínez-Soto et al., 2013). Another study showed that images that included vegetation and sky produced beneficial effects such as improved spatial cognition and a pleasurable sense of motion (Pati et al., 2014).

Besides the studies above that mainly focused on regions of interests (ROIs) in the brain, researchers have also explored the neural connectivity associated with exposure to different environments with fMRI. One study identified ROIs as well as conducted connectivity analysis while participants were viewing images with different vegetation density. Neural activity in the human ventral posterior cingulate cortex (vPCC) changes in response to urban green-space density. Responses in vPCC were significantly higher for intact versus scrambled images, with the greatest differences (intact-scrambled) at the medium density of tree canopy. The cingulate responses are also engaged early in the processing cascade, impacting the attentional and executive regions in a predominantly feedforward manner (Chang et al., 2021). In another study, researchers have observed increases in functional connectivity (FC) between the dorsal attention network (DAN) and the ventral attention network (VAN), FC between the DAN and the default mode network (DMN), as well as FC between the DMN and the Somatomotor when participants were exposed to natural versus built environments (Kuhn et al., 2021). These studies provided evidence that exposure to nature impact brain functioning but also shed little light on the extent to which exposure to various forms of UGI impacts the neural activities associated with different forms of attention.

**Figure 1:**
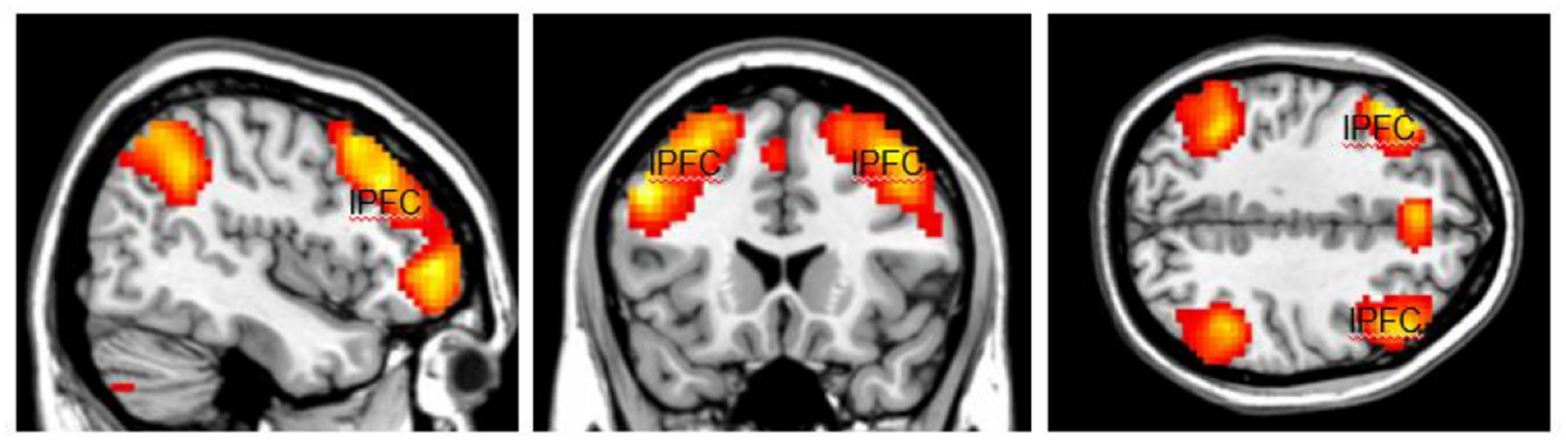
The highlighted areas are the Fronto-Parietal Control Network (FPCN), which includes the lateral prefrontal cortex (lPFC) and posterior parietal cortex in both hemispheres. The FPCN is involved in attentional control and regulates a variety of cognitive processes. Color should be used in print.

**Figure 2:**
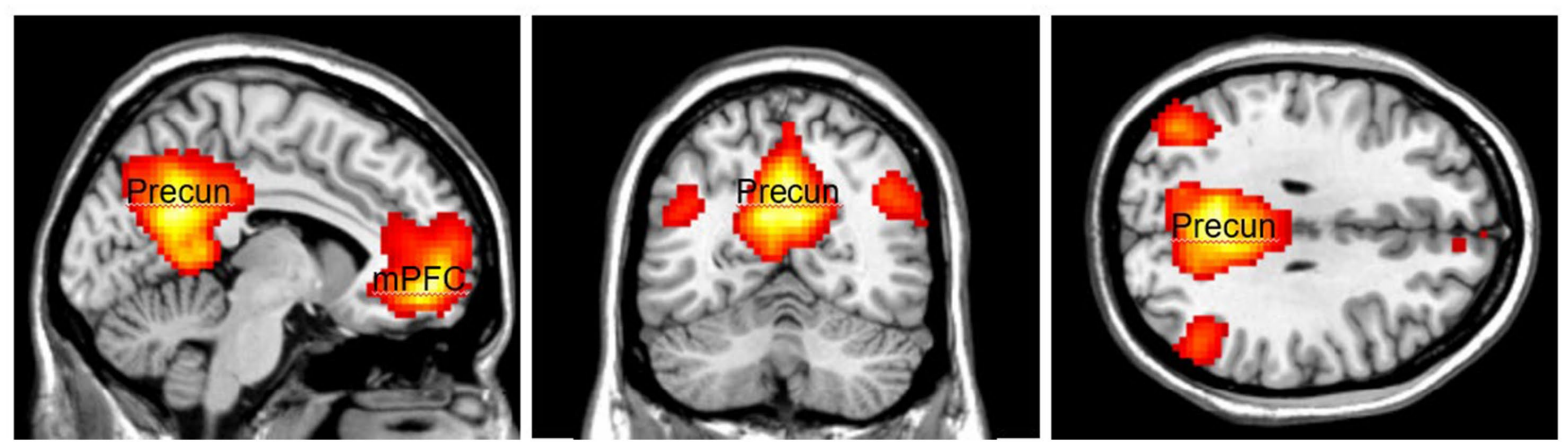
The Default Mode Network (DMN) consists of the medial Prefrontal Cortex (mPFC) and the Precuneus (Precun). DMN is often coupled with the FPCN during attentionally demanding tasks. Color should be used in print.

The activation area associated with top-down attention is often found in the frontoparietal control network (FPCN), which includes the lateral prefrontal cortex (lPFC) and posterior parietal cortex (Figure 1). The FPCN is critical for our ability to coordinate behavior in a rapid, accurate, and flexible goal-driven manner (Marek and Dosenbach, 2018). It is involved in attentional control and regulates a variety of cognitive processes (Cole et al., 2014; van Nieuwenhuizen et al., 2018; Lin et al., 2019). The IPFC controls multiple cognitive processes, such as working memory, attentional selection and planning (Wallis et al., 2015). The FPCN also has extensive brain-wide connectivity; it communicates with a variety of systems throughout the brain (Cole et al., 2010, 2014). For example, the FPCN can inhibit the DMN when it is irrelevant to task performance (Shulman et al., 1997; Chen et al., 2013). The DMN includes regions such as the medial prefrontal cortex (mPFC), precuneus (precun), posterior cingulate and inferior parietal lobes and is often influenced by the FPCN during attentionally demanding tasks (Shulman et al., 1997; Buckner et al., 2008; Maillet et al., 2019; Figure 2).

**Figure 3:**
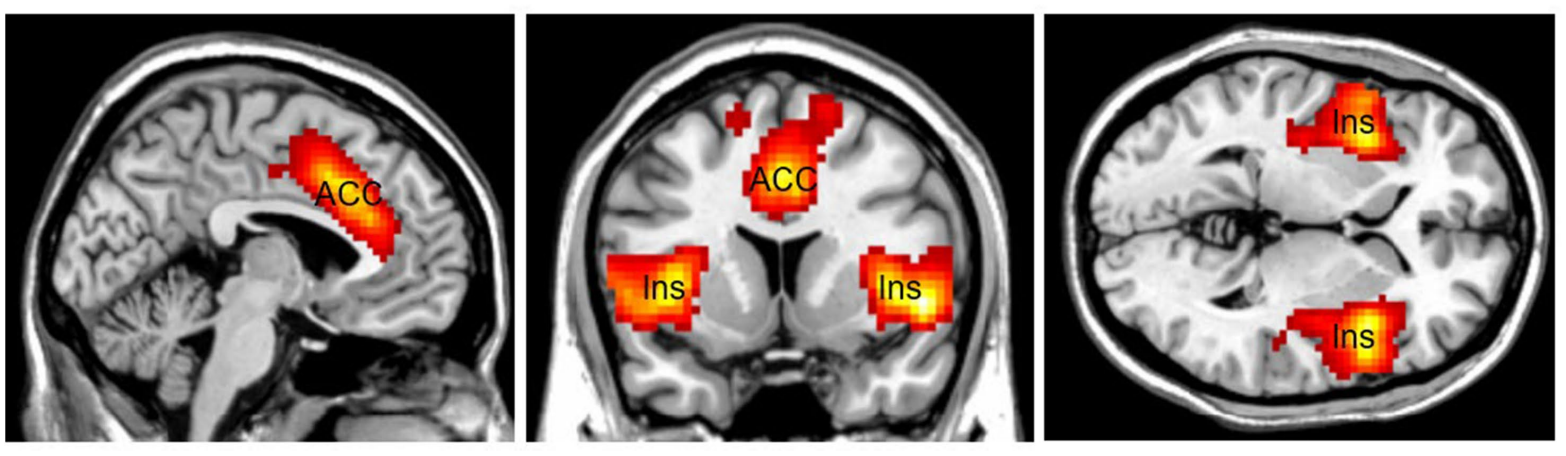
The Salience Network (SN) mainly consists of the Anterior Cingulate Cortex (ACC) and the bilateral insular cortex (Ins). SN is lifesaving by providing vigilance to external stimuli. Color should be used in print.

Another area related to top-down attention is the Salience Network (SN; Figure 3). The SN is a large brain network that includes the dorsal anterior cingulate cortex (dACC), the thalamus, and the anterior insula. Together with its interconnected brain networks, the SN contributes to a variety of complex brain functions, including high-level cognitive control, attentional processes, communication, and social behavior (Craig and Craig, 2009; Menon and Uddin, 2010; Gogolla et al., 2014). The SN is involved in bottom-up attentional processes by providing vigilance, a lifesaving characteristic for human beings. Increasing connectivity has been found between the SN and the DMN for preparatory motor activity and visual motion processing (Clemens et al., 2017). For example, the sound of a fire alarm is an external stimulus that attracts bottom-up attention and requires top-down attention to follow up. The SN increases connectivity with other networks throughout the brain to cope with an emergency. The SN makes people alert and vigilant, helping them survive in emergencies. The SN can work with other brain networks to (1) detect exogenous stimuli, and (2) facilitate access to top-down attention resources when a salient event is detected.

Overall, the FPCN and the SN have been implicated in domain-general processes involved in sustaining attention and cognitive control that support high-level cognitive functioning and trigger prompt reactions to stimuli that seem threatening (Spreng et al., 2010; Maillet et al., 2019).

In the study presented here, we use fMRI to identify FC among the FPCN, the SN, and other networks when participants are exposed to green infrastructure. FC is defined as the temporal synchronicity of geographically dispersed neurophysiological processes (Friston et al., 1996). FC demonstrates the statistical relationship between the measures of activity recorded for two brain regions. Researchers who explore FC observe the extent to which activity in one brain region is temporally correlated with activity in another region. They then compare the neural correlations that emerge as people are exposed to different conditions while they complete a given task.

Increased connectivity of the FPCN is associated with better attentional control and cognitive processes, while decreased connectivity of the SN is associated with less vigilance, which helps sustain top-down attention or promote its recovery in individuals who have fatigued it. If exposure to nature does indeed restore top-down attention, we anticipate that, during exposure to nature stimuli, the neural connectivity associated with the SN will decrease in intensity during, indicating less vigilance, and thus providing an opportunity to restore top-down attention. Simultaneously, we expect to see an increase in neural connectivity associated with the FPCN, indicating more active top-down attention sustained, or restored. Moreover, we expect the opposite to occur for people exposed to urban environments with no green infrastructure: An increase in intensity of connectivity in the SN (indicating greater vigilance), and a decrease in connectivity of the FPCN (indicating decreased top-down attention) during video watching (Table 1).

**Table 1:** The impacts we expect from exposure to green infrastructure in urban environments on neural correlates.

Impacts on neural correlates associated with	Urban environments with green infrastructure	Urban environments without green infrastructure
FPCN	Increase	Decrease
SN	Decrease	Increase

We hypothesize positive association between neural correlates involving FPCN (while watching videos) and exposure to green infrastructure. We also hypothesize a negative relationship between exposure to green infrastructure and neural correlates involving the SN (while watching videos).

We explore the extent to which functional connectivity observed while participants are watching videos that contain varying levels of green infrastructure are consistent with objective tests of attention (SART) to validate our findings.

Our experiment used a randomized controlled design to examine three research questions: (1) To what extent do trees or the combination of trees and bioswales differ in promoting attention restoration? (2) What are the neural correlates in the attention restoration process that result from exposure to different types of green infrastructure? (3) To what extent are the neural correlations in the attention restoration process consistent with actual attention scores (using the Sustained Attention Response Test) from participants viewing different types of green infrastructure?

## 4. Methods

### 4.1. Research site and design

To address the questions posed above, we conducted an experiment in Taipei City, Taiwan. Human Subject approvals were obtained beforehand. We took pictures in Taipei City and selected 24 pictures of urban street settings. We selected pictures that were similar to settings that Taipei residents’ experience in their daily living environments, which include both busy streets and pedestrian-friendly streets.

**Figure 4:**
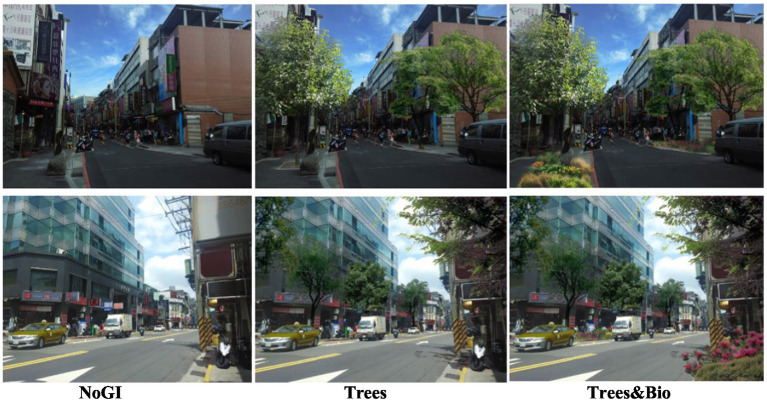
Example images from the NoGI, Trees, Trees & Bio categories (from left to right). Color should be used in print.

After selecting the 24 initial images, we used Adobe Photoshop to remove any vegetation from each image, and in doing so, created the first category: No Green Infrastructure (No GI). Next, we used Photoshop to add street trees to the 24 No GI images to create a second category: Trees. We added tree species commonly found in Taipei. Finally, we added vegetated bioswales to the 24 Trees images to create a third category: Trees & Bio. These three categories (No GI, Trees, and Trees & Bio) have identical physical settings and buildings but vary in the type of green infrastructure that they present (Figure 4). In Taipei City, street trees are prevalent. In some spacious areas, the combination of trees and vegetated bioswales is also often evident. Few streets in Taipei have only bioswales and no trees. Due to the high costs of fMRI experiments, we decided to include the most common GI types (No GI, Trees, and Trees & Bio) in this study and excluded a treatment that included only bioswales.

We designed a between-group experiment to test effects by exposure to videos that included three types of urban green infrastructure. Participants were randomly assigned to one of the three groups. We recorded their neural activity through fMRI while they took the initial SART, while they watched a treatment video, and while they took the second SART. We expected to find variance across the three treatment groups in the before and after differences in SART and FC during video watching.

### 4.2. Participant recruitment

We posted recruitment materials on a local online forum and provided 500 New Taiwan Dollars as an incentive to participate (about 18 U.S. dollars). The recruiting materials stated that the research protocols were approved by our university’s Ethical Review Boards. Forty-three residents of Taipei City (25 males and 18 females) participated in the experiment (Table 2). We aimed to recruit healthy adults between the ages of 20 and 50. In addition to having typical levels of hearing and eyesight, our inclusion criteria required that participants be able to distinguish between the colors red and green. We also required that participants not to: (1) be pregnant or lactating at the time of the test; (2) have a body part with a permanent metal implant, such as artificial joints; (3) have an implanted cardiac pacemaker or any other drug injector; (4) have any temporary metal in the body, such as braces or dentures; and (5) have body tattoos.

**Table 2:** Demographics in three groups.

	No GI	Trees	Trees and bioswales	Average age	Total no.
Male	6	10	9	27	25
Female	8	6	4	25	18
Total	14	16	13	26	43

We determined the sample size by following the procedures from highly cited fMRI studies. In a review that evaluated sample size in neuroimaging research, 96% of highly cited experimental fMRI studies had a single set of research participants, with a median sample size of 12 individuals (Szucs and Ioannidis, 2020).

#### 4.2.1. Experimental procedures

Participants showed up on their assigned day and were informed of the potential risks associated with the experimental procedures. Participants were told that the research objective was to explore brain activity associated with urban settings, but they were not told anything about our hypotheses. They were also made aware of their right to quit the experiment at any time or whenever they felt uncomfortable. They were told that researchers would check their status between steps and would only continue with the experiment if they agreed. Next, participants signed the consent form. Each participant spent between 30 and 40 min in the MRI scanner for the entire experimental procedure.

Upon arrival at our laboratory, each participant rested for 5 min alone. We asked them to remove all rings, watches, credit cards and other devices that might interfere with the MRI scanner. They were scanned for interfering objects before they went into the room with the MRI scanner. Lab staff fixed some stabilizing equipment to their heads to prevent movement during the experiment. Staff also offered participants the opportunity to wear earplugs. A safety ball was placed next to the participants’ dominant hand. They could squeeze the ball to stop the experiment if they were uncomfortable. The experiment progressed through the following steps:

Step 1: We began with a structural scan of the participant’s brain. During these initial scans, participants were encouraged to relax and close their eyes.

Step 2: Next, while participants were still in the scanner, they took the first Sustained Attention Response Test (SART), which lasted for 5 min. During this task a random number from 0 to 9 in different font sizes flashed in the center of the screen, and participants were instructed to click a button with the index finger of their dominant hand when they noticed that the color of the number changed from black to grey. They were also instructed that they should not click the button if the number was 3.

Step 3: To simulate exposure to green infrastructure in this laboratory experiment, participants watched a five-minute video of a slide show that included the 24 images associated with the group to which they had been randomly assigned. The images were presented with a slow zoom in or out from the center of the image. While participants were watching the video, their brains were continuously recorded.

Step 4: After watching the video, participants stayed in the scanner and took the second SART while the scans continued.

Step 5: After participants finished the fMRI part of the experiment, they left the scanner and rested in another room for 5 min.

**Figure 5:**
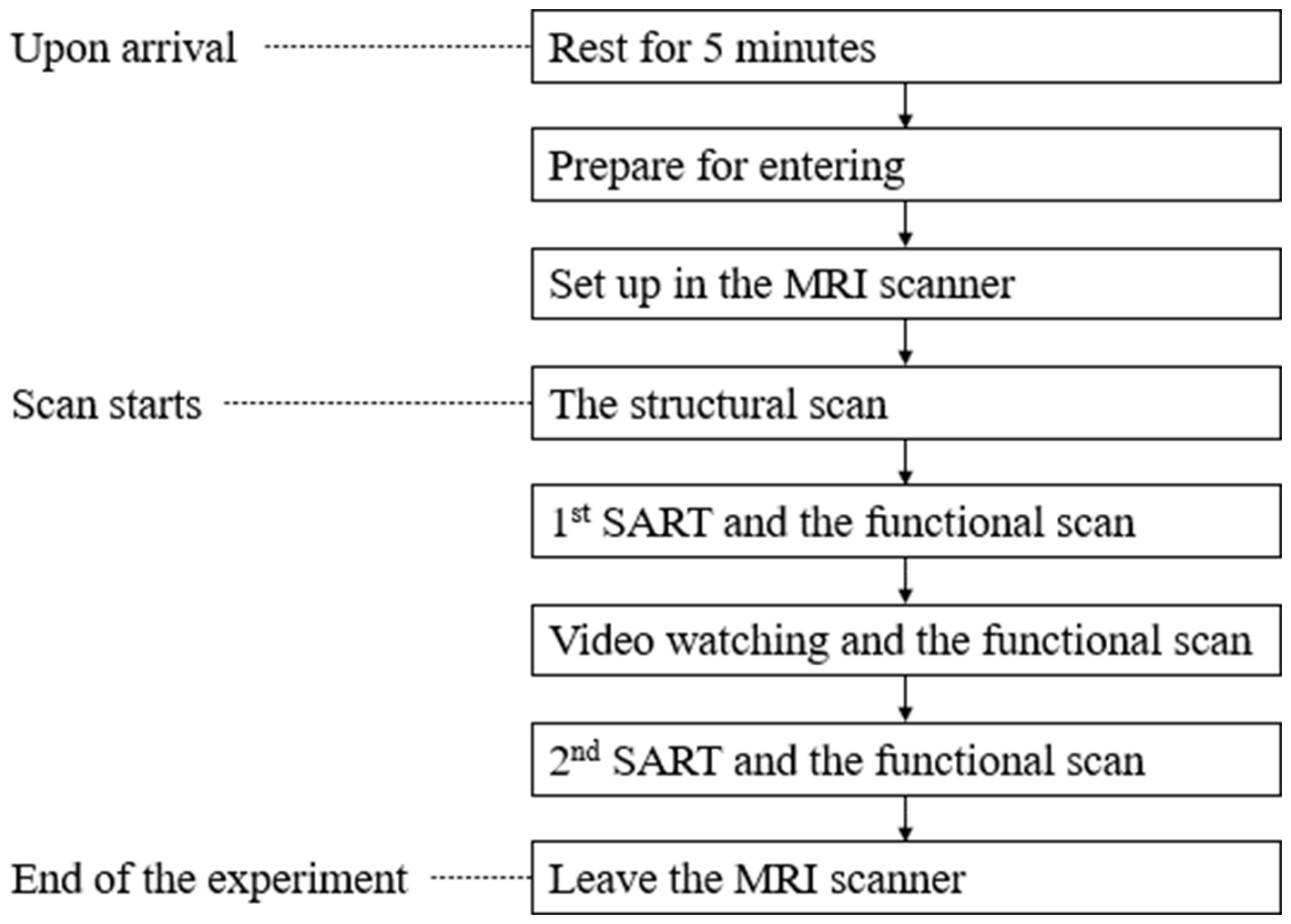
Experimental procedures.

The experiment procedure is depicted in Figure 5.

### 4.3. Construct and measures

The objectives of the research were to clarify the mechanism through which nature restores top-down attention and to observe attentional functioning in action. The independent variable was exposure to different types of green infrastructure, presented through three videos. The dependent variables included participants’ attentional test scores and functional connectivity.

#### 4.3.1. Exposure to green infrastructure

To simulate exposure to green infrastructure in this laboratory experiment, participants were randomly assigned to watch one of the three videos: urban streets with either No GI, Trees, or Trees & Bioswales. The only variation among the three groups was the type of green infrastructure included in the images.

#### 4.3.2. Sustained attention to response test

We employed the Sustained Attention to Response Test (SART) to measure participants’ attentional capacity. SART is a simple, controlled and replicable measure of accuracy and reaction time (Manly and Robertson, 2005). When completing a SART test, participants respond to frequent “go” stimuli, but withhold responses to rare and unexpected ‘no-go’ stimuli (Robertson et al., 1997). In this study, we recorded both accuracy and reaction time, which are believed to be strong predictors for lapsing attention (Manly et al., 2000).

#### 4.3.3. fMRI data acquisition

Scanning was conducted on a 3 T Siemens PRISMA scanner at National Taiwan University. After the localizer, T1-weighted (TR = 2,000 ms; TE = 2.3 ms; flip angle = 8°; FOV = 240 × 240 mm^2^; matrix size = 256 × 256 mm^2^; slice thickness = 0.93 mm; volume size = 192 slices; voxel size = 0.93 × 0.93 × 0.93 mm^3^) and T2-weighted (TR = 9,530 ms; TE = 103 ms; flip angle = 150°; FOV = 192× 192 mm^2^; matrix size = 288 × 384 mm^2^; slice thickness = 3 mm; volume size = 45 slices; voxel size = 0.66 × 0.50 × 3 mm^3^) anatomical images were acquired. Six blocks of full-brain 5-min EPI functional images were acquired axially, coplanar with the AC-PC (TR = 3,000 ms; TE = 30 ms; flip angle = 90°; FOV = 192× 192 mm^2^; matrix size = 64 × 64 mm2; slice thickness = 3 mm; volume size = 45 slices; voxel size = 3 × 3 × 3 mm^3^; number of volumes = 100). Dummy scans (about 11 s) were acquired for signal stabilization and were automatically dropped at the scanner.

#### 4.3.4. Functional connectivity preprocessing

All image preprocessing was done in SPM12 (7487). Functional images first underwent slice timing to temporally align each slice to the start of each volume, and then rigid body realignment was applied to correct for head movement within and across runs using a two-pass procedure (i.e., *via* a mean functional image). To obtain atlas transformation of functional data, an indirect procedure of co-registration and normalization was employed. First, to align functional and anatomical scans, T2-weighted scans were co-registered to the mean functional image and were then used as the reference for co-registration of T1-weighted scans. Next, the aligned T1-weighted scans were submitted to the Segment algorithm in SPM12 to obtain tissue probability maps and deformation fields to be used in normalization. Finally, functional images were resampled in the MNI space on an isotropic 3 mm grid combining realignment and atlas transformation in a single interpolation.

After preprocessing, further steps were taken to denoise the data, including (1) demeaning and detrending across each run, (2) nuisance regression of variables across runs, which used a combination of motion regressors (6 realignment parameters and their first-order derivatives), aCompCor regressors (signals from top 5 principal components generated from each of the tissue maps of white matter and cortico-spinal fluids, along with their first-order derivatives), main condition effects (runs convolved with a canonical hemodynamic response function to further remove simple task-related co-activation confounds), and (3) a simultaneous bandpass filter of [0.009, 0.08] hz. Scrubbing was not implemented in this data due to the short scan duration and consequent small degrees of freedom. Denoising steps were implemented in the CONN toolbox [v. 18b; (Whitfield-Gabrieli and Nieto-Castanon, 2012)] in SPM12.

After denoising, quality control metrics [e.g., framewise displacement (Power et al., 2014)] were calculated and inspected, to assure that data was properly prepared.

#### 4.3.5. Functional connectivity analysis

Brain functional connectivity is a pattern of anatomical links, statistical dependencies, and causal interactions between distinct units within a nervous system (Sporns, 2007). Studying brain connectivity is crucial for understanding how neurons and neural networks process information. Connectivity analyses provide a deeper understanding of the functional interaction among various brain regions than localized brain activation (Adhikari, 2014). As attentional functioning is involved in multiple brain regions and networks, such as FPCN and SN, the analysis of functional connectivity is appropriate as the main analytical strategy. In this study, we aim to demonstrate the phasic changes in the attentive states during the video treatments across the three groups. Previous research has also focused on the phasic changes in neural activity modulated by external stimuli. For example, Herrmann et al. (2016) investigate phasic changes in ROIs and connectivity patterns in amygdala and the bed nucleus of the stria terminalis (BNST) modulated by threatening sound. Unlike sustained responses in the brain, phasic changes in neural activity enable us to understand the influence triggered by external stimuli within the very limited window. Thus, exploring the phasic changes in FC during the video treatment will help us understand the effects of different green infrastructure treatments on attentional functioning. Therefore, we eventually applied *a seed-based functional connectivity analysis* based on clearly identified seeds (e.g., lateral prefrontal cortex and salience network) in relevant literature.

The CONN network atlas, defined by the Human Connectome Project (Whitfield-Gabrieli and Nieto-Castanon, 2012) was used to extract mean signals from an anterior cingulate cortex [ACC; (0, 22, 35)] region of interest (ROI) from the salience network and lateral prefrontal cortex [lPFC; (−43, 33, 28) in the left and (41, 38, 30)] in the right) ROIs from the fronto-parietal control network in both hemispheres. Specifically, we extracted seed time-series from the unsmoothed data aggregated across all voxels within each seed ROI, and voxel timeseries were extracted from the smoothed data at each voxel. We then calculated FC values as the Pearson correlation coefficients (*r*) between the selected seeds and voxels in the rest of the brain, which were then Fisher-transformed into Z scores to allow subsequent statistical testing. All statistical testing was done with age and gender included as covariates of no interest. Pearson’s r would vary between +1 and − 1, where +1 is a perfect positive correlation, and − 1 is a perfect negative correlation. A score of 0 indicates no linear correlation. We extracted other ROIs in the fronto-parietal control network and salience network as seeds too, but ACC and lPFC are the focus of the seed analysis in this study due to the nature of their functioning.

At the second level, to test whether there were differences in seed-based FC patterns among the groups viewing No GI, Trees and Trees & Bio, a general linear model was constructed, with groups being a between-subject factor. Age and gender were entered into the model as covariates of no interest. An F-contrast was used to test for differential seed-based FC patterns between any two groups. Statistical significance was set at a voxel-level threshold of *p* < 0.001 (uncorrected) combined with an extent threshold of *p* < 0.05 (FDR-corrected). To further compare group differences, average connectivity values from significant clusters were extracted and followed up with pairwise tests using Tukey’s procedure.

## 5. Results

We randomly assigned the 43 participants to watch one of the three videos. Fifteen participants were assigned to the No GI group, 16 were assigned to the Trees group and 12 were assigned to the Trees & Bio group. All participants lived in Taipei City and ranged in age from 20 to 44 years old (age average = 26, std. = 5.1). There is no significant difference in age (*p* = 0.71) or gender (*p* = 0.36) between the three groups. All participants reported that they were healthy with no history of mental illness or brain injury.

### 5.1. Attentional tests

We employed the Sustained Attention Response Test (SART) to evaluate participants’ attentional functioning. To understand the extent to which varying types of green infrastructure differ in promoting attention restoration, we compared the accuracy and reaction time differences of the before and after tests between groups. The results of descriptive statistics are reported in Table 3. Group comparisons of the performance on the initial SART tests found no significant differences in attentional functioning among the three groups (Table 4).

**Table 3:** Accuracy and reaction time average in before and after tests.

	Before test average				After test average			
Group	Accuracy		Reaction time		Accuracy		Reaction time	
	Mean	Std	Mean	Std	Mean	Std	Mean	Std
No GI	0.95	0.06	205.49	83.31	0.92	0.11	195.51	87.95
Trees	0.87	0.11	189.40	109.25	0.88	0.12	175.41	87.99
Trees & Bio	0.90	0.08	238.22	94.21	0.90	0.10	234.66	86.72
Total	0.93	0.08	208.64	94.21	0.92	0.10	198.74	86.72

**Table 4:** One-way ANOVA of accuracy and reaction time in the before and after tests.

		Sum of squares	df	Mean square	*F*	Sig.
Before test accuracy	Between groups	0.03	2	0.01	2.01	0.15
	Within groups	0.25	40	0.01		
	Total	0.28	42			
Before test reaction time	Between groups	19837.34	2	9918.67	1.12	0.34
	Within groups	352902.7	40	8822.57		
	Total	372,740	42			

To explore the extent to which different video treatments impacted attention scores, we compared the differences of before test and after test of the SART measures across the three groups. We conducted one-way ANOVAs with the video category as the between-subject factor and found that the treatments had a significant impact on accuracy but not reaction time (Table 5).

**Table 5:** One-way ANOVA of the difference of before test and after test on accuracy and reaction time.

			Accuracy					Reaction time		
	Sum of squares	df	Mean square	*F*	Sig.	Sum of squares	df	Mean square	*F*	Sig.
Between groups	0.02	2	0.01	3.07	0.05	2.56	2	1.28	0	1
Within groups	0.12	40	0			61478.35	40	1536.96		
Total	0.13	42				61480.92	42			

**Figure 6:**
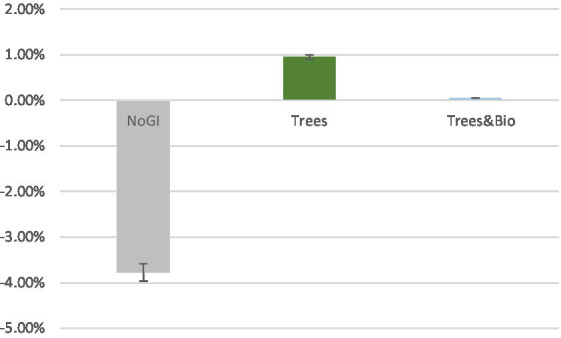
Mean of accuracy change between the two SART tests across the three Groups (No GI, Trees, Trees & Bio). Color should be used in print.

To examine the extent to which different videos impacted accuracy, we made pairwise comparisons using Dunnett *t*-tests, in which the No GI category was the control group, and compared means with the other two groups (Trees and Trees & Bio) (Dunnett, 1980). The difference of accuracy between before and after SART tests demonstrates that participants in the No GI category had the greatest decrease in attention scores (Table 3). The mean decrease in attention score for the No GI group was significantly greater than the Trees group (*p* = 0.02). The Trees & Bio group saw a marginally significant improvement over the No GI group (*p* = 0.06). There was no significant difference in the before and after tests accuracy between the Trees and Trees & Bio groups (Table 6; Figure 6).

**Table 6:** Comparison of mean scores of the change of accuracy between No GI with Trees and Trees & Bio.

(I) VideoType	(J) VideoType	Mean difference (I-J)	Std. error	Sig.	95% Confidence interval	
					Lower bound	Upper bound
Trees	No GI	0.05*	0.02	0.02	0.01	0.09
Trees & Bio	No GI	0.04	0.02	0.06	0.00	0.08

*The mean difference is significant at the 0.05 level.

a Dunnett *t*-tests treat one group as a control and compare all other groups against it.

### 5.2. Network functional connectivity

To what extent did the three treatments impact functional connectivity? To answer this question, we conducted a seed-based analysis for the regions of interest. After analyzing functional connectivity while participants watched their video, we identified two significant connectivity pairs at whole-brain corrected levels in the dorsal lateral prefrontal cortex (dlPFC) and the anterior cingular cortex (ACC; pFDR <0.5; Table 7).

**Table 7:** Significant connectivity pairs during video watching.

Seeds	Clusters	MNI coordinates			F_peak	Z_peak	Cluster size
		*x*	*y*	*z*			
ACC	R Middle occipital gyrus	30	−93	12	12.54	3.82	34
rlPFC	R Middle occipital gyrus	36	−63	30	13.08	3.9	51

**Figure 7:**
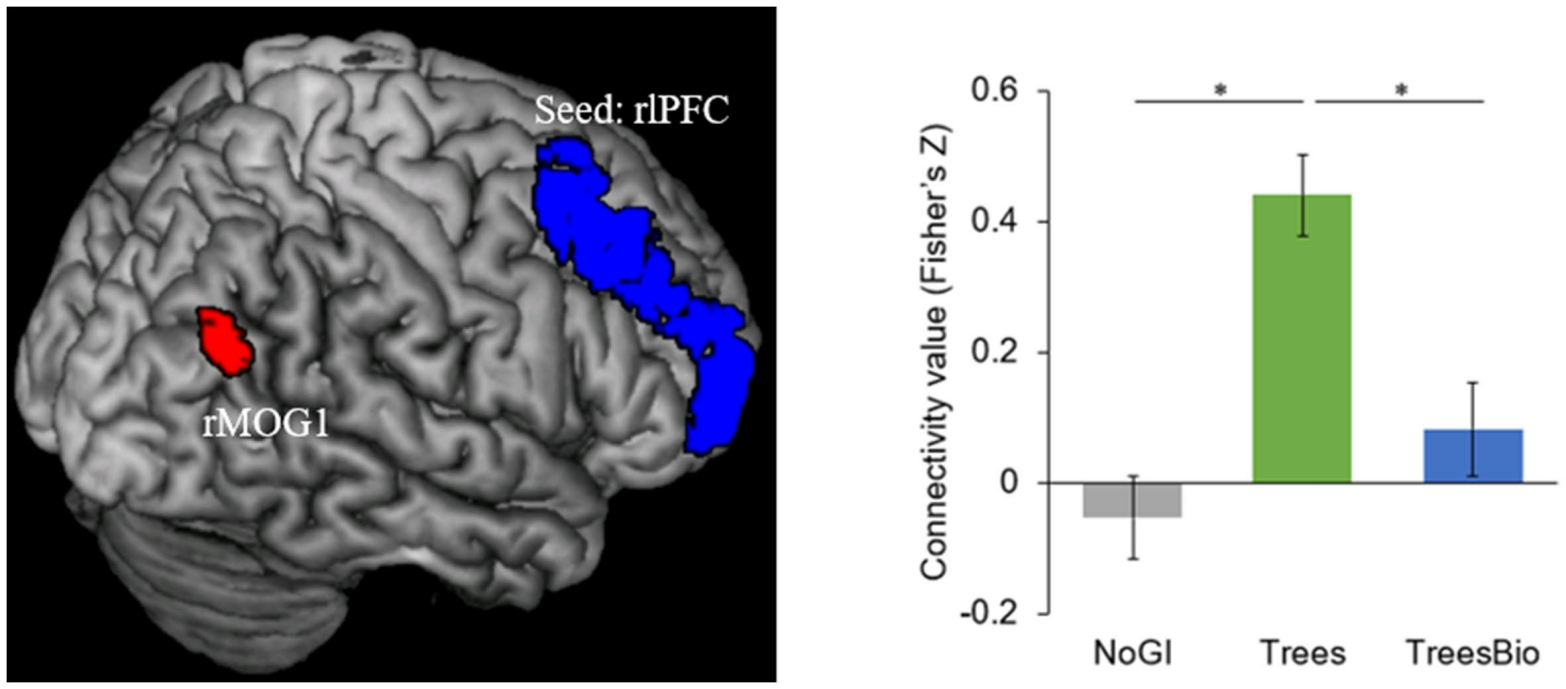
dlPFC and Visual Connectivity during Video Watching. rlPFC-rMOG1 connectivity was significantly higher in the Trees group compared to the No GI group and Trees * Bio group. Color should be used in print.

The first of the two significant functional connectivity levels we identified is between the right lateral prefrontal cortex (rlPFC) and the occipital cortex. During video watching, seeding from rlPFC, F-contrast identified a cluster in the right middle occipital gyrus (rMOG, MNI coordinates [*x*, *y*, *z*] = 36, −63, 30) whose connectivity with rlPFC varied as a function of green infrastructure types in the videos (Figure 7). *Post hoc* Tukey tests showed that the rlPFC-rMOG1 connectivity was significantly higher in the Trees group compared to the No GI group (mean estimate = 0.52, 95% CI = [0.30, 0.74], *p* < 0.0001) and the Trees & Bio group (mean estimate = 0.35, 95% CI = [0.11, 0.58], *p* = 0.002).

The rlPFC is involved in cognitive processes and attentional control. FC associated with it was observed when participants were watching treatment videos and coupled with activation in the occipital regions. As top-down attention is a more advanced function than visual capacity, rlPFC can take control over the occipital regions. As can be seen in Figure 6, participants in the Trees group had the best attentional functioning status when they were watching the video, while participants in the No GI group had the worst. These results are consistent with the behavioral test results in which the Trees group had improved SART performance while the No GI group had decreased SART performance. The relationship between FC associated with FPCN and SART results is positive because better sustained top-down attention can maintain the performance in SART.

**Figure 8:**
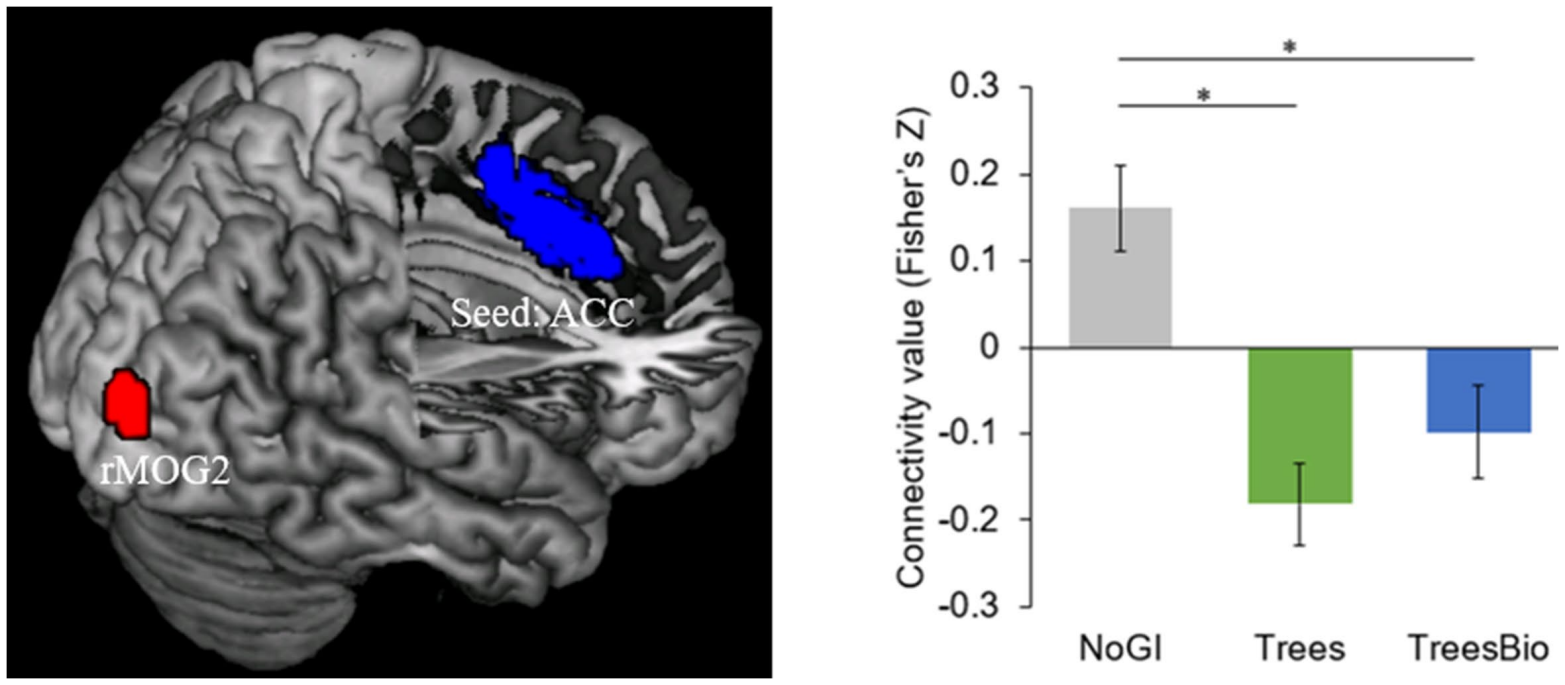
ACC and Visual Connectivity during Video Watching. ACC-rMOG2 connectivity was significantly higher in the No GI group compared to the Trees group and Trees * Bio group. Color should be used in print.

The second significant level of functional connectivity is between the anterior cingular cortex (ACC) and a cluster in the right middle occipital gyrus (rMOG; MNI coordinates [*x*, *y*, *z*] = 30, −93, 12) (Figure 8). The average connectivity values (Fisher’s Z scores) were extracted from this cluster for each subject and were modeled using a one-way ANOVA with the treatment group as a between-subject factor with three levels, and age and gender as covariates. Pairwise *post hoc* tests revealed that the ACC-rMOG2 connectivity in the No GI group was significantly higher than in the Trees group (mean estimate = 0.33, 95% confidence interval (CI) = [0.17,0.50], *p* < 0.0001) and in the Trees & Bio group (mean estimate = 0.25, 95% CI = [0.06,0.43], *p* < 0.01).

ACC is part of the Salience Network (SN), which helps keep people alert. Being alert is an important feature that enables human survival. The SN is involved in the activation of attention as a response to stimuli from the environment. We found that participants in the No GI group had the most active functional connectivity of ACC, while participants in the Trees group had the least active functional connectivity of ACC. The FC associated with SN is negatively related to the SART results because the higher intensity of FC associated with SN represents more vigilance, which may decrease the performance in SART.

As we have identified two pairs of FC associated with the occipital regions, it is important to understand the difference between rMOG1 and rMOG2. rMOG1 is in the visual association area, which oversees processing of advanced visual information. The FC of rlPFC-rMOG1 forms a top-down processing stream that adjusts the pathway of perception according to the task goal. On the other hand, rMOG2 is in the primary visual area, which is responsible for low-level visual information. The FC of ACC-rMOG2 forms a bottom-up processing stream that can detect salient external stimulus (e.g., movement in the environment) and pass that information through the low-level visual cortex. This bottom-up pathway may compete for more attentional resources and act as a circuit breaker. For example, if the external stimulus is a fire alarm, top-down attention will be engaged after the bottom-up processing stream detects the emergency.

### 5.3. Attention scores and functional connectivity

To what extent are the neural correlations consistent with the actual attention scores from participants who viewed different green infrastructure types? To answer this question, we need to refer to the SART scores. After watching the video, participants who had seen the video with trees had the best improvement in SART. The mean change in attention scores (from the first to the second SART) for the Trees Group was significantly greater than that of the No GI Group (*p* = 0.02). The mean change in attention scores for the Trees & Bio Group is not significantly greater than the No GI Group.

Our results suggest that what we see happening in the functional connectivity during the treatment videos is consistent with the results in the changes in the corresponding attention scores. Participants in the Trees group had the greatest positive intensity in functional connectivity between the right lateral prefrontal cortex (rlPFC) and the occipital cortex as well as the greatest negative intensity in functional connectivity between the anterior cingular cortex (ACC) and a cluster in the right middle occipital gyrus. In contrast, participants in the No GI group had the lowest intensity in functional connectivity between the right lateral prefrontal cortex (rlPFC) and the occipital cortex along with the greatest positive intensity of the functional connectivity between the anterior cingular cortex (ACC) and a cluster in the right middle occipital gyrus. Participants in the Trees & Bio group are not different from the No GI group in functional connectivity intensity between the right lateral prefrontal cortex (rlPFC) and the occipital cortex, but they did display a significant difference from the No GI group in the functional connectivity intensity between the anterior cingular cortex (ACC) and a cluster in the right middle occipital gyrus.

To what extent is there a relationship between the two pairs of FC identified in section 6.2 above and the difference of the pre and post attentional tests? That is, do levels of functional connectivity predict changes in scores on the SART tests? To answer this question, we calculate the Pearson correlation coefficient of the FC of rLPFC-rMOG1 and the accuracy improvements in SART and found a correlation of 0.33 (*p* < 0.05). Thus, there is a positive relationship between FC of rlPFC-rMOG1 and the accuracy difference of the before and after tests. The Pearson correlation coefficient of the FC of ACC-rMOG2 and the accuracy improvements in SART is −0.11 (*p* = 0.28). The relationship between FC of ACC-rMOG2 and accuracy improvements is not only insignificant but also weak.

## 6. Discussion

This is the first study that demonstrates a connection between exposure to urban green infrastructure and the neural networks that underline people’s capacity to pay attention. In this study, 43 residents of Taipei City were randomly assigned to one of three groups: No Green Infrastructure, Trees, or Trees & Bioswales.

Participants exposed to the video of urban settings with trees displayed neural activity associated with sustained top-down attentional functioning while watching the treatment video; their corresponding SART results verified that their attention was maintained. Participants exposed to the video of urban settings with trees and bioswales displayed some neural activity indicative of attention restoration, but their SART results did not show significant improvement in attentional functioning. In contrast, individuals exposed to the video of urban environments without green infrastructure displayed neural activity associated with increased vigilance, indicating that attention was not restored; their SART results likewise showed reduced attentional functioning after watching the video. Overall, the SART results support our interpretation of FC associated with FPCN and SN. The consistency between fMRI and SART results provides empirical evidence to support the mechanism of Attention Restoration Theory and demonstrates the efficiency of exposure to trees in supporting attention functioning.

In the paragraphs that follow, we describe the major contributions of this research study, discuss their implications for health-promoting green infrastructure design, list limitations, propose future research opportunities, and draw a short set of conclusions.

### 6.1. Contributions

The main contribution of this study is to identify the neural correlates of attention restoration in response to exposure to urban green infrastructure. These findings add to our understanding of Attention Restoration Theory (ART), which proposes that exposure to nature can help restore top-down attention. Using functional magnetic resonance imaging (fMRI), we found that exposure to urban landscapes with trees resulted in increased functional connectivity in the Fronto-Parietal Control Network (FPCN), a brain region involved in top-down attention and cognitive processes, as well as decreased functional connectivity in the Salience Network (SN), a brain region involved in vigilance. In addition, the results of Sustained Attention to Response Task (SART), a measure of attention, further support the validity of our fMRI results. Overall, our combined SART and fMRI results indicate that exposure to trees can act as a “circuit breaker” to improve attentional capacity by maintaining top-down attention and reducing vigilance.

The second contribution of this study is the addition of new knowledge about the attention restoration benefits of the combination of trees and bioswales. We found that participants in the Trees & Bio group did not have a significant change in attentional test scores and their functional connectivity of the FPCN did not improve, while their functional connectivity in the SN decreased, suggesting a decrease in vigilance rather than an improvement in attention. In contrast, participants in the Trees group had a significant increase in functional connectivity in the FPCN, implying an improvement in attentional functioning. On the other hand, participants in the No GI group had a significant decrease in attentional scores compared to the Trees group, and their functional connectivity in the SN increased, indicating that their top-down attention did not have the opportunity to be restored. These results suggest that adding bioswales to trees may reduce the effectiveness of trees in promoting attention restoration, a finding that has not been reported previously.

Moreover, we have identified two pairs of FC that play different roles in attentional functioning. The FC of rlPFC-rMOG1 forms a top-down processing stream, while the FC of ACC-rMOG2 forms a bottom-up processing stream. When we focus on the No GI group and Trees group, it is obvious that FC of rlPFC-rMOG1 is positively associated with the SART results, while the relationship between the FC of ACC-rMOG2 and SART results is negative. Therefore, participants in the Trees group had better cognitive control which helps to maintain their top-down attention. Participants in the No GI group were more salient, and thus their top-down attention did not get restored. The salience activated by external stimuli, such as the urban settings without GI, was detected by bottom-up attention first. However, if these external stimuli did not have at least some of the four characteristics defined by ART, they do not promote top-down attention restoration, even if the bottom-up attention has been triggered first.

### 6.2. Implications

In this study, we compared the effects of exposure to urban landscapes with varying types of green infrastructure (e.g., trees, understory vegetation (bioswales), and no green infrastructure) on attentional functioning. In the simulations we presented, the appearance of the trees and the bioswales differed from each other in terms of height and plant form. In previous studies, it has been unclear regarding the extent to which bioswales, which consist of understory vegetation similar to rain gardens, contribute to human health. Some research suggests that people do not prefer rain gardens or bioswales that are perceived as wild or unkempt (Appleton, 1975; Kellert, 2002). Other studies, however, have found that heterogeneous landscapes with variations in height can have positive effects on health outcomes (Donovan et al., 2019). Trees and understory vegetation (e.g., flower beds) can increase the perception of being away and eventually contribute to higher ratings of restoration likelihood (Lindal and Hartig, 2015). Our previous research on people’s daily living environments and health outcomes has indicated that understory vegetation is generally associated with negative mental health outcomes (Jiang et al., 2020). In addition, researchers have also found that open grass may increase the incidence of social loneliness (Astell-Burt et al., 2022). These studies show the controversy over the effects of understory vegetation in promoting human health. Future studies may continue to explore the effects of understory vegetation on human health.

Our SART results showed that settings with trees and understory vegetation did not promote attention restoration compared to either the Trees group or No GI group. Although the functional connectivity analysis suggested that adding understory vegetation to trees may decrease functional connectivity involving the prefrontal cortex, the functional connectivity associated with the Salience Network (SN) did not indicate an increase in vigilance. It is important to note that the SART results for the Trees & Bio group were not significant, thus we cannot explain the results of the functional connectivity findings in terms of behavioral our behavioral outcome — the SART. The inconsistency between SART and functional connectivity outcomes corresponds with the debate about the effects of understory vegetation on attention restoration. Further empirical evidence is needed to help us understand how understory forms of green infrastructure are related to human health.

Green infrastructure, such as bioswales and rain gardens, is often introduced in urban landscapes as an ecological solution to environmental challenges such as stormwater runoff. The effects these green infrastructure on attentional functioning, however, are often overlooked. The evidence presented here can serve as a starting point for future guidelines on the use of various types of green infrastructure, particularly in densely populated cities where space is limited and green infrastructure like bioswales may be used in courtyard and roof gardens to provide vegetation and biodiversity. It is important to consider the potential effects on attentional functioning when implementing these types of green infrastructure.

### 6.3. Limitations and future research

Neuroimaging advances have allowed us to examine the effects of various forms of green infrastructure on psychophysical outcomes. Analyzing functional connectivity in the brain has provided insights into nature’s impacts attention, supporting Attention Restoration Theory. Still, interpreting neural activity is complex due to the human brain’s multifaceted nature and concurrent neural functions. For example, perceiving soft fascination from nature can involve both top-down and bottom-up attention, and the trade-offs between these attentional functioning types may fluctuate rapidly. Identify regions of interest (ROIs) or networks solely associated with one type of functioning is challenging.

Many researchers have begun to investigate nature’s effects on neural activity in various brain regions, exploring patterns of brain activation while participants view natural and urban landscapes (Kim G.-W. et al., 2010; Kim T.-H. et al., 2010; Norwood et al., 2019). Researchers have also identified ROIs for bottom-up attention associated with exposure to various landscape (Tang et al., 2017). With a deeper understanding of brain function and functional connectivity, more studies have employed neural connectivity analysis (Chang et al., 2021). Our investigation, based on prior research, identified novel functional connectivity when participants were exposed to different UGI. Future interdisciplinary research involving scholars with expertise in landscape architecture, urban planning, psychology, and neuroscience is needed to deepen our understanding of how nature can promote mental health.

To ensure the validity of our findings, we randomly assigned participants to one of three groups, carefully controlled for potential confounding variables, and continuously recorded participant’s responses to stimuli using empirical scans. It is worth noting, however, that like previous empirical studies (Lee et al., 2015; Deng et al., 2022), our research involved participants viewing videos of urban green infrastructure rather than interacting with real physical landscapes. Real-world interactions involve walking through, smelling, and hearing environments. Future research should explore how incorporating these sensory experiences to fMRI studies influences results. Additionally, we did not consider participants’ daily routines, daily exposure to green infrastructure, or physical activity, which can also impact attentional functioning (Li et al., 2018; Zhang et al., 2018) and cognitive functioning (Li and Sullivan, 2016; Wells, 2000). Future research might record neural activity over a longer time frame or in a task-unconstrained condition so that we might understand how green infrastructure in real-world settings impacts attentional functioning in daily life.

We used the SART test to measure participants’ attentional functioning. SART, however, was originally developed for individuals with brain injuries and may not be as challenging for others. Using more difficult attentional tests may reveal more noticeable differences between groups who have been exposed to different landscape treatments. Additionally, SART is administered at a very fast pace – one trial per second – which makes it difficult to conduct event-related analysis using fMRI scans with a time resolution of 3 s per image. Future research could use more challenging tests of attention that are administered at a slower rate or for a longer duration.

The results of the Trees & Bio group suggest that adding bioswales to landscapes with trees may reduce the effectiveness of trees in restoring attentional functioning. Yet, due to the lack of significant differences in SART scores between the Trees & Bio and Trees groups, as well as the lack of significant difference between Trees & Bio and No GI, we cannot conclude that Trees & Bio do not promote attention restoration or that trees are not affected by the presence of bioswales. As mentioned in the research design section, there are not many streets in Taipei with bioswales but no trees, so we did not create a set of simulations for bioswales alone due — a decision driven the high cost of conducting fMRI experiments and the lack of practical scenarios in Taipei. In the future, it would be useful to test the effects of understory vegetation on attention restoration and explore newer green infrastructure forms’ impact on attention restoration’s neuro-underpinnings. Researchers could also examine different green infrastructure combinations on psychological outcomes. By demonstrating the health benefits of specific green infrastructure combinations, we can apply these findings to landscape and urban design encouraging wider adoption of such interventions.

## 7. Conclusion

This study is, to the best of our knowledge, the first to employ fMRI to investigate the relationship between exposure to urban green infrastructure and attentional functioning. By examining the impacts of exposure to various forms of green on neural functional connectivity during attention restoration, we have found support for the mechanism proposed by Attention Restoration Theory (ART). Our findings represent a crucial step toward understanding how nature influences brain functioning in terms of attention restoration and contribute to our understanding of ART’s hypothesized mechanism.

Both the behavioral results and functional connectivity data indicate that viewing scenes with trees can sustain better top-down attention compared to viewing scenes without green infrastructure. We also found that adding bioswales to settings with trees diminished the benefits provided by trees alone. This suggests caution should be exercised when implementing newer types of green infrastructure, even if they offer additional ecological benefits such as animal habitat, increased biodiversity, and flood reduction. The results presented here can provide evidence for city administrators, policy makers, and other stakeholders interested in urban greening initiatives, providing them with greater confidence in creating greener, healthier places for the people they serve.

## Data availability statement

The data presented in the study are deposited in the Baidu Netdisk. The link of the repository is: https://pan.baidu.com/s/11ToTCne3T5BUSHzGKOTvew. The accession numbers are: 0314.

## Ethics statement

The studies involving human participants were reviewed and approved by Research Ethics Committee, National Taiwan University; Institutional Review Board (IRB), University of Illinois at Urbana-Champaign. The patients/participants provided their written informed consent to participate in this study.

## Author contributions

XJ: conceptualization, methodology, software, validation, formal analysis, investigation, resources, data curation, writing – original draft, writing – review and editing, visualization, supervision, and project administration. YH: conceptualization, methodology, software, validation, formal analysis, data curation, writing – original draft, and visualization. LL: writing – review and editing. C-YC and WS: conceptualization, writing – review and editing, supervision, project administration, and funding acquisition. All authors contributed to the article and approved the submitted version.

## Funding

This work is funded by (1) *Landscapes on the Brain*. USDA Forest Service, North Central Research Station. Funding code: 569 AG 15-JV-11242309-105; (2) Ministry of Science Technology, Taiwan. Funding code: MOST 104-2410-H-002-207-MY2; (3) *Talent Introduction Program*. Xihua University. Funding code: Z212029.

## Conflict of interest

The authors declare that the research was conducted in the absence of any commercial or financial relationships that could be construed as a potential conflict of interest.

## Publisher’s note

All claims expressed in this article are solely those of the authors and do not necessarily represent those of their affiliated organizations, or those of the publisher, the editors and the reviewers. Any product that may be evaluated in this article, or claim that may be made by its manufacturer, is not guaranteed or endorsed by the publisher.
